# Modeling analysis of the dynamics of thrombocytopoietic, granulocytopoietic and erythropoietic systems in irradiated humans

**DOI:** 10.1093/jrr/rrt150

**Published:** 2014-03

**Authors:** Olga Andreevna Smirnova

**Affiliations:** Federal State Unitary Enterprise Research and Technical Center of Radiation-Chemical Safety and Hygiene, 40 Shchukinskaya St, Moscow 123182, Russian Federation

**Keywords:** blood, bone marrow, humans, modeling, radiation effects

## Abstract

Biologically motivated mathematical models, which describe the dynamics of the thrombocytopoietic, granulocytopoietic and erythropoietic systems in irradiated humans, are developed and thoroughly investigated [ 1– 3]. These models are implemented as the systems of non-linear ordinary differential equations, whose variables and constant parameters have clear biological meaning. The modeling studies revealed general regularities and characteristic features of the dynamics of the aforementioned hematopoietic lineages in acutely and chronically irradiated humans. It is shown that the modeling predictions qualitatively and quantitatively agree with the respective clinical data for humans exposed to acute and chronic irradiation in wide ranges of doses and dose rates. The ‘lethal’ dose rate of chronic irradiation, which is evaluated in the framework of the granulocytopoiesis model, coincides with the real minimal dose rate of lethal chronic irradiation for humans. As for the thrombocytopoiesis and erythropoiesis models, the respective ‘lethal’ dose rates of chronic irradiation are very close to the real one for humans. All this bears witness to the validity of employment of the developed models in the investigation and prediction of radiation effects on human hematopoiesis. These models could provide a better understanding of the risks to health from the space radiation environment for astronauts on long-term space missions. The models could also be helpful in estimating the hazards for health of a population, which resides in contaminated areas after an accident.
